# Concurrent paclitaxel-based chemo-radiotherapy for post-surgical microscopic residual tumor at the bronchial margin (R_1_ resection) in non-small-cell lung cancer

**DOI:** 10.1186/s12885-015-1036-0

**Published:** 2015-02-06

**Authors:** Meixiang Zhou, Tao Li, Yongmei Liu, Changjin Sun, Na Li, Yong Xu, Jiang Zhu, Zhenyu Ding, Yongsheng Wang, Meijuan Huang, Feng Peng, Jin Wang, Li Ren, You Lu, Youling Gong

**Affiliations:** 1 Department of Thoracic Oncology, Cancer Center and State Key Laboratory of Biotherapy, West China Hospital, Sichuan University, Chengdu, 610041 P.R. China; 2 Radiation Physics Center, Cancer Center, West China Hospital, Sichuan University, Chengdu, 610041 P.R. China; 3 Department of Radiation Oncology, Second People’s Hospital of Sichuan, Chengdu, 610031 P.R. China; 4 Department of Oncology, Second Affiliated Hospital of Anhui Medical University, Hefei, 230601 P.R. China

## Abstract

**Background:**

The microscopic residual tumor at the bronchial margin after radical surgery (R1 resection) affects prognosis negatively in non-small-cell lung cancer (NSCLC) patients. For patients with good performance status, a potential cure still exists. Here, we report the outcomes of concurrent paclitaxel-based chemo-radiotherapy (CRT) for NSCLC patients with microscopically positive bronchial margins or peribronchial infiltration.

**Methods:**

A retrospective search in the clinical database was conducted in three hospitals. Patients were identified and evaluated if treated with radiotherapy combined with paclitaxel-based chemotherapy. The objects analyzed were local control time, progression-free survival (PFS), overall survival (OS), and treatment-related toxicity.

**Results:**

Sixty-one patients with microscopic residual tumor at the bronchial stump following pulmonary lobectomy were identified. Forty-six patients who had received concurrent paclitaxel-based CRT were analyzed. The median follow-up was 40 months (range: 15.0–77.5 months). The 1-, 2- and 3-year survival rates were 97.8%, 60.9% and 36.9%, respectively. The local recurrences were recorded in 19.6% (9/46) patients. Median PFS and OS for the evaluated cohort were 23.0 [95% confidence interval (CI): 21.3–24.7] and 32.0 (95% CI: 23.7–40.3) months, respectively. The most common side effects were hematological toxicity (neutropenia, 93.5%; anemia, 89.1%; and thrombocytopenia, 89.1%) and no treatment-related deaths. Grade ≥2 acute radiation-induced pneumonitis and esophagitis were recorded in 43.5% (20/46) and 26.1% (12/46) patients, respectively. By univariate analysis, non-squamous cell lung cancer was associated with a significantly longer survival time (45.1 *vs* 26.4 months, *p* = 0.013).

**Conclusions:**

For NSCLC patients with post-surgical microscopic residual tumor at the bronchial stump, concurrent paclitaxel-based chemo-radiotherapy achieved promising outcomes with accepted treatment-related toxicity.

## Background

Anatomic pulmonary lobectomy with radical lymph node dissection is the primary treatment for operable non-small-cell lung cancer (NSCLC) [1]. Complete resection of NSCLC should be confirmed pathologically when all resection margins are free from tumor (R_0_ resection). The incidence of microscopic residual tumor at the bronchial margin (R_1_ resection) is 4–5% (range: 1.2–17%) of all lung operations [2]. Although the classification of an R1 resection at the bronchial margin is not uniform in the literature, Wind *et al.* concluded that it could be divided into submucosal residual disease, peribronchial residual disease, and extrabronchial residual disease [2]. Microscopic residual tumor might negatively affect prognosis, with 1- and 5-year survival rates among these patients between 20-50% and 0–20%, respectively [2]. So far, there have been no randomized trials comparing different treatment strategies in such patients. Nevertheless, the panel of the National Comprehensive Cancer Network (NCCN) still recommended that repeat resection or chemo-radiotherapy should be considered if the patients have positive bronchial margins [1]. In such patients, a potential for cure still exists.

Liewald *et al.* reported that in patients after R_1_ resection, reoperation might improve survival in Stage I (64 *vs* 21 months) and Stage II (38 *vs* 12 months) disease [3]. Snijder *et al.* reported 28 patients with Stage I NSCLC and microscopic residual tumor at the bronchial margin [4]. The 5-year survival rate of the patients who underwent reoperation was 40% as compared with 27% in patients that did not. Therefore, reoperation in patients with Stage I and II NSCLC and R1 resection of the bronchial resection margin is recommended [1,3-5]. Similarly, postoperative radiotherapy (PORT) is often given in clinical practice if microscopic residual tumor is present at the resection margin, based on the results of several retrospective studies showing a reduction in the local recurrence rates [6-8]. However, the value of PORT is controversial and some studies have reported high local recurrence rates following PORT in this specific population [4,9]. Thus, the NCCN panel indicated that CRT is an alternative strategy for Stage II or III disease with bronchial positive margins [1].

In clinical practice, patients with NSCLC after a R_1_ resection at the bronchial margin may be considered as potentially curable if their performance status is good. Concurrent CRT consisting of cisplatin and etoposide, paclitaxel and cisplatin (TP), and paclitaxel and carboplatin (TC) regimens has been used for salvage and definitive treatment, according to the NCCN guidelines [1].

In this study, we retrospectively evaluated the clinical outcomes of patients treated with curative-intent CRT, giving detailed information of the survival and related side effects, with the intention of proving suitable treatment for patients after R_1_ resection at the bronchial margin.

## Methods

### Patient data

R1 resection was defined as invasive microscopic residual tumor at the bronchial margin, or peribronchial infiltration without any tumor lesion at the bronchial stump area at baseline computed tomography (CT) 4 weeks after surgery. Between March 2007 and August 2012, 61 NSCLC patients received CRT for bronchial positive margin at West China Hospital, Second People’s Hospital of Sichuan, and Second Affiliated Hospital of Anhui Medical University. Forty-six patients received paclitaxel-based CRT. All of the patients had histologically proven NSCLC. This retrospective study was carried out with the approval of the Ethics Committee of West China Hospital, the Second People’s Hospital of Sichuan and the Second Affiliated Hospital of Anhui Medical University.

The basic and clinical characteristics of the study population are summarized in Table 1. The median age of the patients was 57 years; most of them were male and had an Eastern Cooperative Oncology Group (ECOG) performance status score of 0–1. Twenty-eight and fourteen patients had squamous cell carcinoma and adenocarcinoma, respectively. The initial tumor stage (Staging system, American Joint Committee on Cancer, 6^th^ edition) [10] after surgery was Stage II (11; 23.9%), Stage IIIa (29; 63.0%), and Stage IIIb (6; 13.1%). The median follow-up was 40.0 months (range: 15.0–77.5 months).

**Table 1:** **Basic and clinical characteristics of the patients in present study (n = 46)**

Characteristics	Number of patients (%)
***Age (years)***	
Median (range)	57 (39–75)
***Gender***	
Male/Female	35 (76.1)/11 (23.9)
***ECOG*** ^***a***^ ***performance status***	
0-1	42 (91.3)
2	4 (8.7)
***Pathology***	
Squamous-cell carcinoma	28 (60.9)
Adenocarcinoma	14 (30.4)
Other types	4 (8.7)
***T staging after surgery*** ^***b***^	
T2/T3/T4	11 (23.9)/21 (45.7)/14 (30.4)
***N staging after surgery*** ^***b***^	
N0/N1/N2	10 (21.7)/22 (47.8)/14 (30.4)
***Tumor stage after surgery*** ^***b***^	
II	11 (23.9)
IIIa	29 (63.0)
IIIb	6 (13.1)
***Follow-up time (months)***	
Median (range)	40 (15.0-77.5)

^*a*^: Eastern Cooperative Oncology Group; ^*b*^: Staging system, 6^th^ edition, American Joint Committee on Cancer, 2002.

### Concurrent CRT

#### Paclitaxel-based chemotherapy

The regimens consisted of paclitaxel 175 mg/m^2^ plus cisplatin 75 mg/m^2^ (TP regimen), paclitaxel 175 mg/m^2^ plus or carboplatin (AUC = 5) (TC regimen), or paclitaxel 175 mg/m^2^ plus oxaliplatin 135 mg/m^2^ (TO regimen) on Day 1 given every 3 weeks. The only adverse effects were Grade ≥3 acute treatment-induced pneumonitis or esophagitis, and if prolonged, chemotherapy was discontinued. Otherwise, the chemotherapy was suspended until recovery and the drug dose was reduced by 25% in the subsequent cycle.

According to the NCCN guidelines [1], adjuvant chemotherapy (including concurrent cycles with radiotherapy) was delivered at a maximum of four cycles.

### Radiotherapy

All patients underwent CT simulation. Gross tumor volume (GTV) was defined as the site of the bronchial positive margin (2 cm around the bronchial stump). As described in Table 2, the clinical tumor volume (CTV) enclosed the GTV with an 8-mm margin and the high-risk draining lymph node stations followed the classification by Mountain *et al.* [11]. For the planning target volume (PTV), a 10-mm margin was added isotropically to the CTV (PTV1) and GTV (PTV2). The dose-volume constraints for the lungs were set as follows: V_20_ < 22% and mean lung dose <12 Gy. A maximum dose of 45 Gy was allowed to the spinal cord (planning risk volume).

**Table 2:** **Lymph node stations** ^***a***^ **irradiated as the CTV** ^***b***^

	N_0–1_after surgery	N_2_after surgery
**Right**		
*Upper/Middle lobectomy*	10,7 and 4R	10, 7, 4R [irradiate 2R if 4R (+)]
*Lower lobectomy*	10 and 7	10, 7 [irradiate 8/9 if 8/9 (+)]
**Left**		
*Upper lobectomy*	10, 7, 4 L and 5	10, 7, 4 L and 5 [irradiate 2 L if 4 L (+)]
*Lower lobectomy*	10 and 7	10, 7 [irradiate 8/9 if 8/9 (+)]

^*a*^: Followed the lymph node classification [11]; ^*b*^: clinical target volume.

The patients received a conventional-fraction schedule. The dose prescribed for PTV1 was 50 Gy and that for PTV2 was at ≥60 Gy. Radiotherapy started at the latest on the first day of the second chemotherapy cycle.

The details of the concurrent CRT are shown in Table 3.

**Table 3:** **Treatment in present study (n = 46)**

Time-interval between resection and start of treatment	
Median/range (weeks)	5/4-6
**Radiotherapy**	
*PTV volume* ^*a*^ *(cm* ^*3*^ *, median/range)*	182.6/162.2-278.4
*Irradiation dose for PTV2* ^*b*^ *(Gy, median/range)*	60/50-70
*Number of fractions (median/range)*	30/25-35
*Total lung V*_*20*_ *(%,median/range)*	21/17-24
*Mean lung dose (Gy,median/range)*	11.7/10.3-12.8
**Chemotherapy**	
*Chemotherapy regimens* ^*c*^	
Paclitaxel and Cisplatin	31 (67.4%)
Paclitaxel and Carboplatin	11 (23.9%)
Paclitaxel and Oxaliplatin	4 (8.7%)
*Number of chemotherapy cycles (median/range)*	3 (1–4)
*Number of concurrent cycles (median/range)*	2 (1–3)

^*a*^: Planning target volume; ^*b*^: generated according to the GTV; ^*c*^: all chemotherapy regimens were delivered per three weeks.

#### Treatment assessment

Local failure was defined as recurrence at the bronchial stump and within the irradiated field. The regional failure was defined as lymph node recurrence outside the irradiated field. Local control was defined as no recurrence in the local and regional fields. Progression was defined as local recurrence or appearance of new lesions. Follow-up evaluations were performed 4 weeks after treatment, every 2–3 months for the first 2 years, and every 6 months thereafter.

Toxicity was evaluated and graded according to the National Cancer Institute Common Toxicity Criteria version 3.0 [12]. A diagnosis of radiation-induced pneumonitis was made on the clinical symptoms (including cough, shortness of breath and fever), with radiological findings in the absence of any other likely cause.

### Statistical methods

Statistical analyses were performed using SPSS version 17.0. Progression-free survival (PFS) was measured from the date the treatment began to the date of disease progression, and overall survival (OS) was considered from the start of treatment to the date of data analysis, or date of loss from follow-up for patients alive, or date of death. Patients without local recurrence or progression who discontinued follow-up for any reason were censored on the last day of tumor assessment. The rates of PFS and OS were calculated using the Kaplan-Meier method. Log-rank test and Cox’s proportional hazards regression model were used for univariate survival analysis. Patient age, sex, ECOG performance status, pathological type, disease stage after surgery, chemotherapy regimen, and radiation dose were included in univariate analysis.

## Results

All patients received a radiation dose of ≥50 Gy (for PTV1), and 78.3% (36/46) patients completed the planned radiotherapy (for PTV2). The median radiation dose delivered was 60 Gy, with a range of 50–70 Gy (Table 2). Thirty-one, 11 and 4 patients received the TP, TC and TO regimens, respectively, and 6.5% (3/46), 89.1% (41/46) and 4.3% (2/46) of patients received one, two and three cycles of chemotherapy with concurrent radiotherapy, respectively.

**Figure 1:**
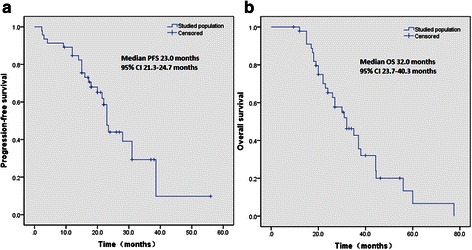
**Kaplan-Meier analysis of progression-free survival (a) and overall survival (b) in the present study.**

Follow-up studies continued until December 2013, with no one lost. Only one patient was diagnosed with brain metastasis at the first follow-up. Local and regional failure was observed in four and two patients, respectively. One patient was diagnosed with local and regional failure. The local control rate was 84.8%. The 1-, 2- and 3-year survival rates were 97.8%, 60.9% and 36.9%, respectively. Median PFS and OS for the evaluated cohort were 23.0 months [95% confidence interval (CI): 21.3-24.7 months) and 32.0 months (95% CI: 23.7-40.3 months), respectively (Figure 1).

### Treatment-related toxicity

All the patients were evaluated for treatment-related toxicity (Table 4). The combination of chemotherapy (either TP, TC or TO regimens) and radiotherapy proved to be tolerable. The most common toxicity was neutropenia (93.5%, 43/46). Grade 3 and 4 neutropenia was observed in 16 (34.8%) and one (2.2%) patients, respectively. Other major toxicities G1/2 included anemia, thrombocytopenia and acute esophagitis. Grade 3 treatment-related acute pneumonitis was observed in three (6.5%), nausea and vomiting in 8 (17.3%) patients. No grade 5 toxicity was recorded among any patients.

**Table 4:** **The treatment-related toxicities in present study (n = 46)**

Toxicities^*a*^	Toxicity grades, n (%)				
	Grade 0	Grade 1	Grade 2	Grade 3	Grade 4
***Hematological***					
Neutropenia	3 (6.5)	12 (26.1)	14 (30.4)	16 (34.8)	1 (2.2)
Anemia	5 (10.9)	25 (54.3)	16 (34.8)	0	0
Thrombocytopenia	5 (10.9)	21 (45.7)	20 (43.4)	0	0
***Non-hematological***					
Nausea and vomiting	9 (19.6)	17 (37.0)	12 (26.1)	8 (17.3)	0
Acute esophagitis	6 (13.0)	28 (60.9)	12 (26.1)	0	0
Acute pneumonitis	8 (17.3)	18 (39.2)	17 (37.0)	3 (6.5)	0

^*a*^: According to the Common Toxicity Criteria for Adverse Events, version 3.0.

### Systemic treatment after disease progression

Twenty-seven patients (58.7%) were recorded with disease progression during follow-up. Local recurrence and tumor metastasis were observed in nine (19.6%) and 18 (39.1%) patients, respectively. Most of them (92.6%) received systemic treatment after disease progression, and only two patients received palliative radiotherapy. Among the patients with squamous-cell lung cancer, eight patients had received the gemcitabine/platinum regimen. Among the patients with non-squamous cell lung cancer, five patients had received the pemetrexed/platinum regimen and 5 patients had received tyrosine kinase inhibitors (TKIs: erlotinib or gefitinib).

### Univariate survival analysis

**Figure 2:**
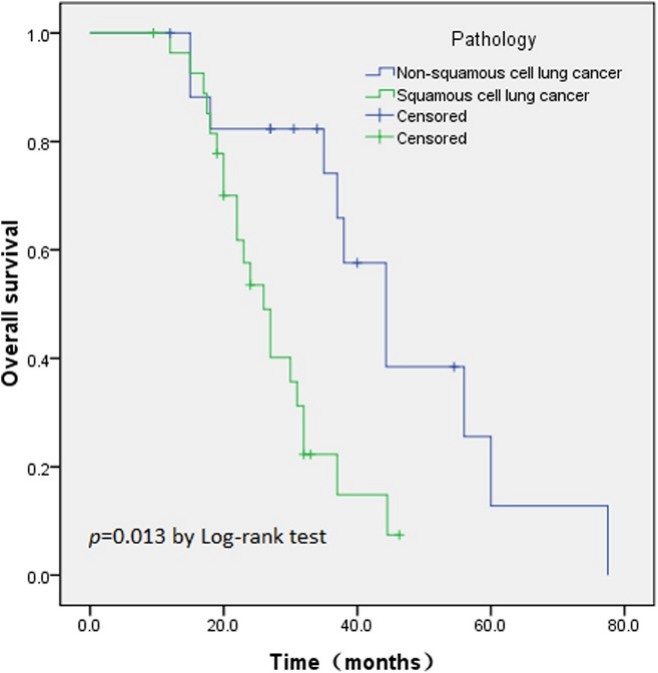
**Kaplan-Meier analysis of overall survival in the present study, according to the pathology type of the patients.**

Because of the small number of patients, only univariate analysis was performed according to the basic and clinical characteristics of the patients. The details are shown in Table 5. Age, sex, ECOG performance status, disease stage after surgery, chemotherapy regimen, and radiation dose did not significantly affect survival time. Pathological type (non-squamous cell lung cancer) was significantly associated with improved OS (median: 45.1 months), compared with patients with squamous-cell lung cancer (median OS: 26.4 months, *p* = 0.013) (Figure 2).

**Table 5:** **Prognostic factors by log-rank test and univariate survival analysis** ^***a***^ **in present study**

Factors	Group	Number	Median OS^*b*^(months)	Log-rank test*p*value	Univariate analysis*p*value
**Age**	<57 years	24	33.2	0.876	0.853
	≧57 years	22	30.8		
**Gender**	Male	35	31.3	0.949	0.932
	Female	11	32.8		
**ECOG** ^***c***^ **performance status**	0-1	41	32.4	0.906	0.887
	2	4	29.6		
**Pathology**	Squamous-cell lung cancer	28	26.4	0.013	0.017
	Non-squamous cell lung cancer	18	45.1		
**Staging after surgery** ^***d***^	II	11	35.2	0.654	0.613
	III	35	30.0		
**Chemotherapy regimen**	TP	31	33.7	0.393	0.308
	TC/TO	15	30.5		
**Irradiation dose**	≧60 Gy	40	33.6	0.505	0.496
	<60 Gy	16	29.3		

^***a***^: Cox’s proportional hazards regression model; ^*b*^: overall survival; ^*c*^: Eastern Corporative Oncology Group; ^*d*^: According to the AJCC 6^th^ staging system.

## Discussion

To our knowledge, this is the first report of concurrent CRT for post-surgical microscopic residual tumor at the bronchial margin in patients with NSCLC. Paclitaxel-based chemotherapy and concurrent radiotherapy achieved a median OS of 32.0 months among those NSCLC patients after R1 resection, with tolerable treatment-related toxicity. Although this was a retrospective evaluation with a small sample, our data suggest that selected NSCLC patients after R1 resection may benefit from aggressive and curative-intent concurrent CRT.

Reports from Liewald *et al.* and Snijder *et al.* show that for Stage I and II NSCLC patients with positive margins, repeat resection could improve OS [3,4] and is recommended by NCCN [1] for Stage II patients. In the present study, 11 patients (23.9%) with Stage II NSCLC did not undergo repeat resection, but received CRT. As recorded in our database, four patients refused reoperation because of limited cardiopulmonary function, and the others did not want a second operation. Among these patients, the median survival time was 35.2 months, which was similar to the reported data of repeat resection in Stage II cases by Liewald *et al.* (median OS: 38 months) [3].

The NCCN panel recommends the concurrent CRT for R_2_ resection or mediastinal recurrence, and the sequential CRT for R_1_ resection [1]. However, there is no direct evidence of any disadvantage of concurrent settings in NSCLC patients with R_1_ resection. Concurrent chemoradiation improves the clinical outcomes of Stage IIIA or IIIB disease [13-15]. In the present study, salvage CRT was well-tolerated and toxicity was as expected from thoracic CRT. The median OS was 32.0 months, which is comparable to those data collected from patients with bronchial stump recurrence [16]. Recently, Bar *et al.* reported the outcomes of CRT for loco-regional recurrence of NSCLC after surgery, and the median survival after recurrence was 26.9 months [17]. Thus, for patients with good ECOG performance status after R_1_ resection, concurrent CRT is still a treatment of choice.

It should be mentioned that three patients (6.5%) developed acute grade 3 radiation pneumonitis after treatment. Two patients had received right lower lobectomy and one left lower lobectomy. The delivered dose was 60, 62 and 60 Gy respectively, and the chemotherapy regimen was paclitaxel and cisplatin. They were diagnosed with Grade 3 radiation pneumonitis between 2 and 4 weeks after radiotherapy and finally recovered after steroid therapy. The incidence of RP is somewhat lower than the concurrent CRT for locally advanced NSCLC. Among several parameters based on dose-volume histograms, V_dose_ and mean lung dose (MLD) are important predictive factors of acute radiation pneumonitis [18,19]. In definitive chemo-radiotherapy for NSCLC, the NCCN panel suggests that a V_20_ value of 30-35% and MLD <20 Gy are thresholds for symptomatic radiation pneumonitis [1]. However, there is little information of such a threshold in the post-lobectomy situation. Uno *et al.* reported that in a 21-patient population, three patients developed grade ≥2 radiation pneumonitis after concurrent CRT [20]. They found that the V_20_ < 20%/MLD <10 Gy might be predictive factors for grade ≥2 radiation pneumonitis in post-lobectomy patients receiving definitive radiotherapy. In our practice, the lung constraints were set as V_20_ < 22% and MLD <12 Gy. All these constraints need more studies for validation.

In this study, the drug doses were modified in 19 (41.3%) patients during treatment. In addition, the granulocyte colony stimulating factor (G-CSF) is routinely applied for secondary prophylaxis in our practice when patients have grade 1 or 2 neutropenia in the preceding cycle. In some situations, we use G-CSF prophylactically among patients after chemotherapy to avoid any break in radiotherapy. So, the rate of grade 4 neutropenia (1 patient, 2.2%) was lower than expected. No grade ≥3 acute esophagitis was observed in this study. To avoid any break in radiotherapy as a consequence of grade 3 acute esophagitis, we usually prescribe a liquid combination (500 ml 0.9% physiological saline injection, 10 ml 1% lidocaine injection, and 10 mg dexamethasone mixed with each other, 15 ml P.O three times per day) among the patients with acute grade 2 esophagitis.

Another issue that should be discussed here is the target delineation. As all tumors are microscopic at the bronchial margin, it was difficult to define the GTV. Griess *et al.* [21] and Cotton [22] have reported that even after resection, in which there is a macroscopic tumor-free margin >2 cm, the incidence of R_1_ resection was still around 6% among these resections. From the report by Olszyna-Serementa *et al.*, 80 patients with R_1_ resection have been analyzed [23]. They concluded that the PORT results in a relatively better survival in these patients. They also suggested that the elective nodal irradiation was useful for local control in pN_0–1_ patients. At present, the precise definition of GTV and CTV in an R_1_-resection situation had not been concluded and needs more clinical investigation.

By univariate survival analysis, we found that pathological type (non-squamous cell lung cancer) was significantly longer survival time, compared with patients with squamous-cell lung cancer (45.1 *vs* 26.4 months, *p* = 0.013). This result differs from the studies of Ghiribelli *et al.* [5] and Liewald *et al.* [3]. This may in part be explained by imbalances in the selection of subsequent treatment regimens with respect to histological subtypes. Currently, it is well known that several new anti-tumor drugs (including pemetrexed and TKIs) could significantly prolong the survival time among patients with metastatic non-squamous cell lung cancer, since a series of landmark trials has been published [24-27]. It might be the reason that these patients survive longer than the others in the present study.

Limitations of the present study should be mentioned. First, the retrospective nature of the study and the small number of the patients must be paid attention when interpreting the results. Second, the patients analyzed in this study had good ECOG performance status (0 or 1). Some patients could not tolerate the adjuvant CRT or chemotherapy alone, if their performance status was 2 or 3.

## Conclusions

Concurrent paclitaxel-based chemo-radiotherapy is a feasible treatment strategy for NSCLC patients after R1 resection, with as-expected treatment-related toxicity. However, the most suitable chemotherapy regimen and the optimal radiotherapy planning (target delineation and normal tissue constraints) are not established and require further investigation.
